# Keeping in time with social and non-social stimuli: Synchronisation with auditory, visual, and audio-visual cues

**DOI:** 10.1038/s41598-021-88112-y

**Published:** 2021-04-22

**Authors:** Juliane J. Honisch, Prasannajeet Mane, Ofer Golan, Bhismadev Chakrabarti

**Affiliations:** 1 Centre for Autism, School of Psychology and Clinical Language Sciences, University of Reading, Reading, RG6 6AL UK; 2 Department of Psychology, Bar-Ilan University, Ramat Gan, Israel

**Keywords:** Motor control, Sensorimotor processing, Sensory processing, Human behaviour

## Abstract

Everyday social interactions require us to closely monitor, predict, and synchronise our movements with those of an interacting partner. Experimental studies of social synchrony typically examine the social-cognitive outcomes associated with synchrony, such as affiliation. On the other hand, research on the sensorimotor aspects of synchronisation generally uses non-social stimuli (e.g. a moving dot). To date, the differences in sensorimotor aspects of synchronisation to social compared to non-social stimuli remain largely unknown. The present study aims to address this gap using a verbal response paradigm where participants were asked to synchronise a ‘*ba*’ response in time with social and non-social stimuli, which were presented auditorily, visually, or audio-visually combined. For social stimuli a video/audio recording of an actor performing the same verbal ‘*ba*’ response was presented, whereas for non-social stimuli a moving dot, an auditory metronome or both combined were presented. The impact of autistic traits on participants’ synchronisation performance was examined using the Autism Spectrum Quotient (AQ). Our results revealed more accurate synchronisation for social compared to non-social stimuli, suggesting that greater familiarity with and motivation in attending to social stimuli may enhance our ability to better predict and synchronise with them. Individuals with fewer autistic traits demonstrated greater social learning, as indexed through an improvement in synchronisation performance to social vs non-social stimuli across the experiment.

## Introduction

Everyday social interactions such as talking with friends, playing games (i.e. pat a cake) or lifting a table together require us to continuously monitor and predict the behaviour of others, and adjust our movements accordingly to achieve smooth interactions. The ability to closely synchronise our movements with external stimuli has been proposed to have evolutionary functions for resource acquisition and reproduction^1^ by facilitating cooperation^2^ and social cohesion^3,4^. One of the most common channels used for synchronous interactions is the verbal channel, as in simple parent–child games, singing, sloganeering or through the entrainment of speech rhythms during everyday conversations^5,6^.

Previous research on sensorimotor synchronisation has largely used finger tapping paradigms to examine synchronous responses to non-social stimuli, such as flashing lights or auditory metronomes^7,8^. However, most stimuli that we synchronise to are social in nature, e.g. the movements of another person, dance, speech or songs. Studies that have investigated synchronising to social stimuli have generally used a different class of paradigms with more ecological validity, e.g. synchronous arm curling^9^, walking together^10^, dance-like movements^11^ or synchronous bouncing^12^. The focus of these studies has been on social affiliative and cognitive outcomes rather than the accuracy of synchronous responses to social stimuli. Due to this historical diversity of experimental approaches in studying synchrony in social and non-social domains, the differences in synchronising to social vs non-social stimuli remain largely unknown. While social compared to non-social stimuli have been shown to be processed differently in multiple studies^13–17^, none of these studies have used synchrony based measures to examine how we physically interact with such stimuli. Most studies in this area use paradigms where observers passively observe social and non-social pictures/videos presented on a screen^14,16–19^ . In contrast, interpersonal synchrony involves an active involvement with the environment, and is more typical of real-world social situations where participants are not merely passive observers.

The real world rarely offers stimuli in a single sensory modality. Accordingly, it is vital to consider cues in single as well as multiple sensory modalities within each class of stimuli (social and non-social). Previous reports using non-social stimuli have shown closer synchronisation performance in terms of mean asynchrony when synchronising to an auditory compared to a visual metronome^20–22^. This finding is not unexpected, as audition has been found to be superior in temporal processing, whereas the visual modality is superior in spatial processing^20^. However, visual dynamic stimuli compared to discrete visual stimuli have been found to improve synchronisation performance^23^. Specifically, in bimodal (audio-visual) stimuli, individuals have been shown to optimally integrate information from both modalities^24^. Elliott, Wing and Welchman^25^ showed that synchronous finger tapping with bimodal discrete stimuli was more accurate compared with unimodal stimuli. However, little is known about the impact of the number of modalities on synchronisation to social stimuli.

Engaging in synchronous interactions facilitates social bonds, by increasing liking^3^, trust^26^, prosocial behaviour^27,28^ and reducing outgroup bias^29^. Individuals diagnosed with autism spectrum disorders (ASD) typically face challenges in social communication and sensory processing^30^, and often experience difficulties in integrating multisensory information^31^. In lab-based studies, autistic individuals show a reduced preference for social compared to non-social stimuli^32–34^. ASD has also been associated with atypical spontaneous facial mimicry and spontaneous motor synchronisation with another person^35–38^. In comparison with typically developing children and adults, individuals with ASD have been found to produce weaker and more variable synchronisation behaviours to social stimuli^39,40^.

The present study aims to systematically examine the differences in synchronisation behaviour to social compared to non-social stimuli. Here, we developed a novel verbal response paradigm in which participants synchronised their verbal response in time with social or non-social stimuli (presented unimodally, audio or visual) or bimodally (audio-visual). We predict that individuals will perform differently in how well they synchronise with social compared to non-social stimuli. One possibility is that individuals will be worse in synchronising to social stimuli due to their greater complexity (i.e., a face is significantly more visually complex compared to a dot). Another possibility is for individuals to show better performance in synchronizing with social stimuli, due to the greater reward value typically attributed to social stimuli^16,19^. The second possibility is supported by empirical and theoretical accounts suggesting greater reward response associated with motor alignment^41–43^. For both social and non-social stimuli, synchronisation with audio-visual combined conditions is expected to result in lower asynchrony compared to the audio and visual only conditions^25^. Further, in line with the literature on auditory superiority when synchronising with a metronome, we predict closer synchronisation with auditory compared with visual stimuli^20–22^.

In this study, autistic traits were measured using the Autism Spectrum Quotient^44^ (AQ). Autistic traits are distributed continuously throughout the population with similar aetiology at both ends, allowing us to examine the impact of autism-related variation at a population level^45^. We predict that autistic traits will be negatively associated with synchronisation performances across tasks, in line with earlier findings of weaker and more variable synchronisation performances in individuals with ASD ^38–40^.

## Results

Model fit statistics for the linear mixed model implemented were as follows:

$${\text{AIC}} = - 1726.84,{\text{ BIC}} = - 1602.964,{\text{R-squared conditional}} = 0.273$$

Results of the linear mixed model analysis are presented below in Tables 1 and 2.

**Table 1:** Parameter estimates for the fixed effect omnibus tests.

Fixed effect omnibus tests				
	F	Num df	Den df	*p*
Condition	16.697	2	2590.7	< .001
Stimulus type	797.117	1	2604.7	< .001
AQ	0.166	1	40.1	0.686
Gender	0.307	1	39.9	0.583
Condition * Stimulus Type	44.432	2	2592.6	< .001

**Table 2:** Parameter estimates for the fixed effects of the model.

Fixed effects parameter estimates								
Names	Effect	Estimate	SE	95% Confidence Interval		df	t	*p*
				Lower	Upper			
(Intercept)	(Intercept)	0.34766	0.0180	0.31248	0.38285	55.0	19.368	< .001
Condition1	C–A	− 0.01649	0.0111	− 0.03831	0.00533	2595.1	− 1.481	0.139
Condition2	V–A	− 0.03573	0.0113	− 0.05788	− 0.01357	2600.9	− 3.161	0.002
Stimulus type 1	Social–Nonsocial	− 0.24283	0.0117	− 0.26571	− 0.21995	2592.0	− 20.804	< .001
AQ	AQ	3.96e−4	9.74e−4	− 0.00151	0.00231	40.1	0.407	0.686
Gender 1	Male-Female	− 0.00565	0.0102	− 0.02564	0.01433	39.9	− 0.554	0.583
Condition 1 * Stimulus type 1	C–A * Social-Nonsocial	0.01112	0.0164	− 0.02111	0.04336	2587.5	0.677	0.499
Condition 2 * Stimulus type 1	V–A* Social-Nonsocial	0.14213	0.0167	0.10937	0.17488	2594.0	8.505	< .001

Conditions are denoted as follows (A: Auditory, C: Combined, V: Visual). Stimulus Types are denoted as Social or Nonsocial.

**Figure 1:**
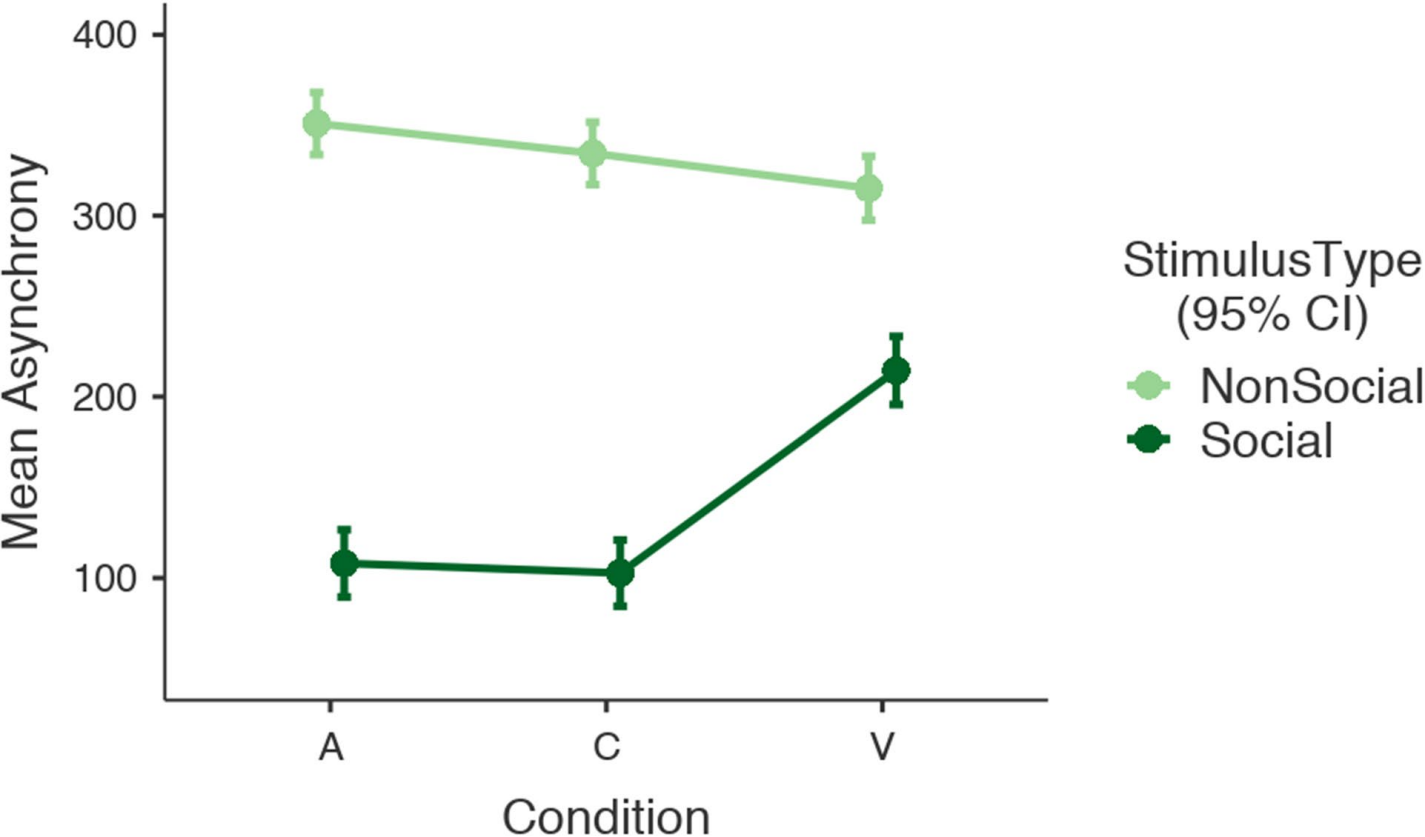
This figure shows the effect of Stimulus Type (Social, Non-social) and Condition (A: Auditory, C: Combined, V: Visual) on mean asynchrony in seconds. The error bars signify a 95% confidence interval.

The model revealed a main effect of Stimulus Type (F(1,2604.7) = 797.12, *p* < 0.001) with social stimuli associated with significantly lower mean absolute asynchrony (estimated marginal mean = 142 ms, 95%CI [129, 154]) compared to non-social stimuli (estimated marginal mean = 334 ms, 95%CI [322, 345]). A main effect of Condition was also observed (F(2,2590.7) = 16.70, *p* < 0.001) with visual stimuli (estimated marginal mean = 265 ms, 95%CI[251, 279]) associated with significantly larger absolute mean asynchrony compared with auditory (estimated marginal mean = 230 ms, 95%CI[216, 243]) and audio-visual combined stimuli (estimated marginal mean = 219 ms, 95%CI[216, 243], see Fig. 1). No significant difference between auditory and audio-visual combined conditions was observed. An interaction effect between condition and stimulus type was noted (F(2,2592.6) = 44.43, *p* < 0.001). All pairwise post-hoc comparisons show that social stimuli were associated with better synchronization performance compared to non-social stimuli, irrespective of sensory modality. For non-social stimuli, participants synchronized better with visual than with auditory conditions [t(2600) = 3.160, *p* = 0.023]. For social stimuli, synchronisation performance was better for auditory and audio-visual conditions in comparison to visual conditions [t_visual-audiovisual_(2584) = 9.156, *p* < 0.001; t_auditory-visual_(2584) = − 8.644, *p* < 0.001]. A full list of post-hoc comparisons is provided in the Supplementary Materials.

No effects of AQ (F(1,40.1) = 0.166, *p* = 0.686) or gender (F(1,39.9) = 0.307, *p* = 0.583) were noted.

It is possible to measure synchrony using either the visual or auditory onsets in response to the combined audio-visual stimulus. Synchronisation performance to social combined (audio-visual) stimuli was further examined to test if it differed as a function of using auditory vs visual onsets to define the target cue. This analysis was restricted to social stimuli alone, since auditory and visual onsets for non-social audio-visual stimuli were programmed to be identical. The results revealed a significant difference in mean asynchrony as a function of onset type [t(2282) = − 10.94, p_Bonferroni_ < 0.001], with higher mean asynchrony associated with the visual onset (estimated marginal mean = 201 ms, 95% CI [264.1,320]) than with the auditory onset (estimated marginal mean = 142 ms, 95% CI [75.4,130]).

**Figure 2:**
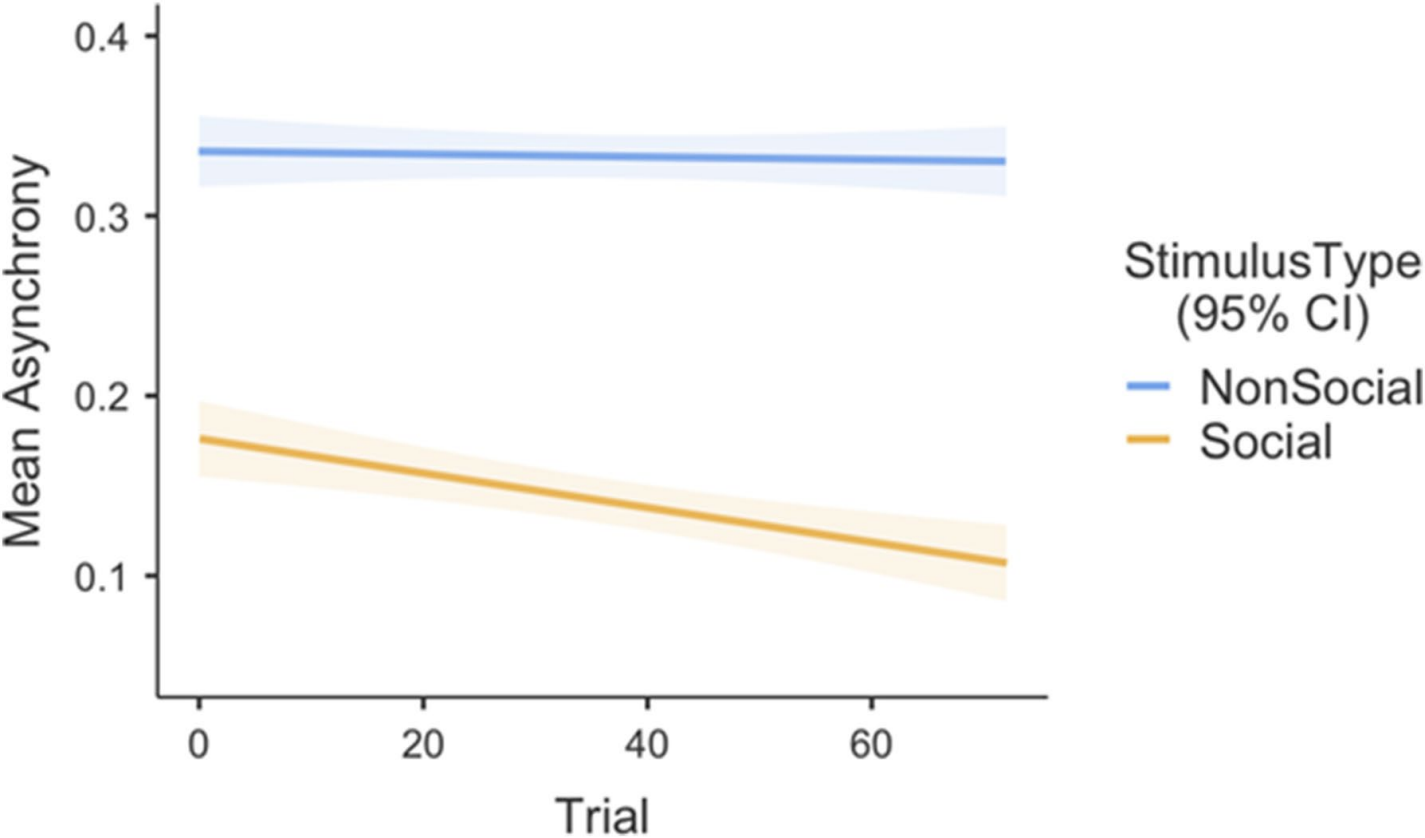
This figure shows the two-way interaction for the learning effect, indicating a decrease in mean absolute asynchrony for social but not for non-social conditions. Stimulus Type (Social, Non-social) on mean asynchrony in seconds. The error bars signify a 95% confidence interval.

**Figure 3:**
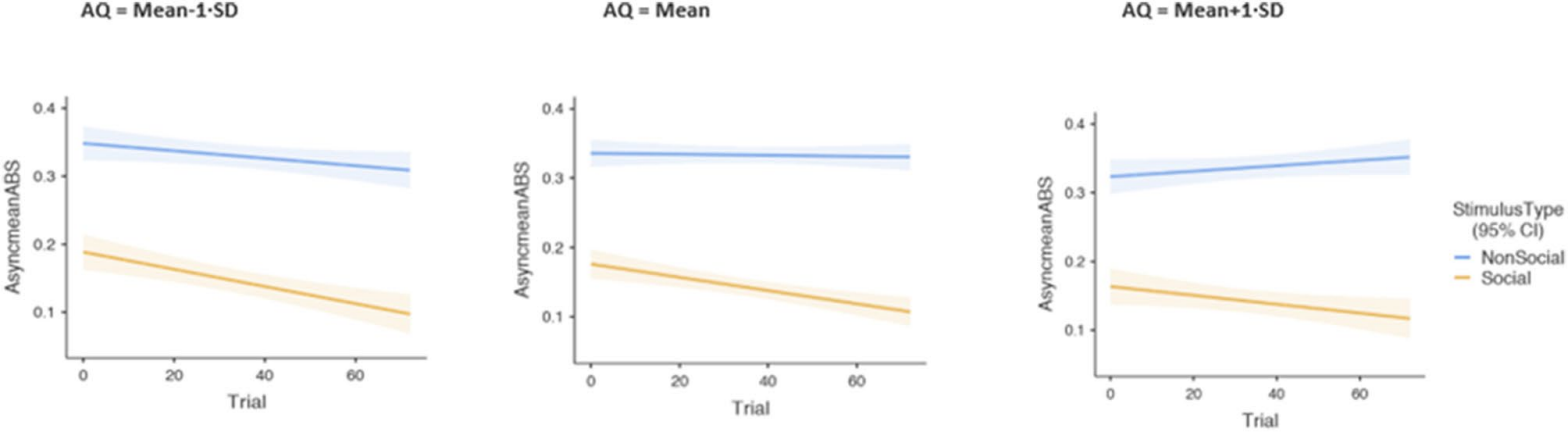
This figure shows the three-way interaction for the learning effect and AQ, indicating a decrease in mean absolute asynchrony across trials for social compared with non-social stimuli. The *left panel figure* illustrates that participants who scored 1 SD below the sample mean showed a steep decrease in mean absolute asynchrony across trials for social compared with non-social stimuli. In contrast, participants who scored 1 SD above the mean AQ scores (*right panel figure*) showed a less steep decrease in mean absolute asynchrony in response to social vs non-social stimuli across trials.

An exploratory analysis to measure learning/practice effects through the experiment revealed a main effect of trial [F(1,2586.2) = 9.942, *p* = 0.002], a significant two-way interaction of trial and stimulus type [F(1,2586.2) = 7.341, *p* = 0.007] as well as a significant three-way interaction between stimulus type, trial, and AQ [F(2,2588.3) = 3.154, *p* = 0.043] (see Figs. 2 and 3).

Figure 2 illustrates the data from the two-way interaction by showing a monotonically decreasing trend of mean absolute asynchrony for social trials, suggesting the existence of a practice/learning effect. This trend is not seen in response to the non-social trials. Figure 3 demonstrates the three-way interaction between stimulus type, trial, and AQ.

## Discussion

The present study systematically tested the differences in synchronising with social and non-social stimuli, when presented in single or multiple sensory modalities. Mean asynchrony for trials in response to non-social stimuli was found to be greater than in response to social stimuli. Additionally, mean absolute asynchrony for visual stimuli were found to be higher than for auditory and audio-visual (combined) stimuli. No effect of autistic traits or gender were noted on mean absolute asynchrony.

Participants were significantly better in synchronising with a social compared to non-social stimuli, across all three conditions (auditory/visual/audio-visual), despite the greater variability in the target cue timings for social stimuli. This finding could potentially be an outcome of increased attention or reward value ascribed to social over non-social stimuli^17,19,34,46^. Infants less than four weeks old have been found to show preferential attention to social stimuli such as human sounds, movements and facial features^47,48^. Increased reward value of target stimuli can influence both the starting point as well as the rate of sensory data accumulation^49,50^. Greater relative reward value for social stimuli can therefore potentially explain closer synchronous performances for such stimuli as observed in our current study. Another plausible explanation for this difference is the use of verbal responses in the present study. Verbal responses match the nature of the social but not the non-social stimuli in our paradigm. The compatibility between the action performed in the social stimuli and the motor response performed by participants may have improved motor simulation by activating the same neural systems involved in perceiving and producing the same motor response^51^. Consequently, participants’ predictive model of the stimuli, along with participants’ motor planning and execution could have been enhanced, resulting in closer synchronous performances^52^. Further, given the social nature of human actions, whether it is making a *ba* sound or a finger tap, it is not entirely possible to decouple response compatibility from the sociality of stimuli. Another possible contributing factor for the improved synchronisation observed for social stimuli could be the lower pitch of the male and female actor in comparison with the higher pitch of the metronome. Synchronisation with auditory metronomes can be influenced by the pitch of the sounds, with better synchronisation associated with lower pitch sounds^53,54^. However, no systematic analysis has been conducted to estimate the magnitude of this effect on a wide range of pitches that are comparable to those used in this study, and whether this finding can be generalised to human vocal sounds.

Our exploratory analysis to check for practice/learning effects revealed a significant difference between the social and non-social conditions. Across the whole sample, individuals tended to perform better (i.e., with less absolute mean asynchrony) as the experiment progressed. This learning effect was greater for social compared to non-social stimuli, which could reflect greater attention being drawn to the dynamic social stimulus (a face saying ‘*ba ba*’) in comparison to the bouncing dot. This social learning effect was greater in individuals with lower autistic traits, than those with higher autistic traits. This observation is consistent with a recent report showing reduced integration of social information in a learning task in individuals with high autistic traits^55^.

The observation of differences in response to social and non-social stimuli raises an important question about the origin of these differences. The low-level properties of social and non-social stimuli used in this study are different (e.g., contrast, colour, nature of sound). It is possible that the distinction between ‘social’ and ‘non-social’ stimuli is a cumulative effect driven by a large number of such low-level properties. Perfect matching for all stimulus properties will render the two sets of stimuli identical to one another. It is worth noting that the category of ‘social’ stimuli represents a circumscribed set of low-level stimulus features (e.g., flesh tones for visual stimuli, sound within the vocal frequency range for auditory stimuli). Whether there is a ‘social’ advantage over and above all potential physical characteristics of the stimulus remains an open question. Although our paradigm has higher ecological validity by presenting a real recording of a human partner, examining synchronisation that replicates a real-life social interaction that involves mutual and reciprocal adaptation would be of interest for future research.

In view of the significant interaction between stimulus type and condition observed in the main analysis, we separately analysed synchronisation performance by stimulus type. For non-social stimuli, there was little difference between the three conditions (see Fig. 1), but participants synchronised better with visual, than with auditory stimuli. This finding is in contrast to a previous study that showed better synchronization of finger tap responses to an auditory compared to a visual stimulus^56^. One potential explanation is that continuous visual stimuli provide a more salient temporal cue to allow for better temporal judgements than discrete auditory stimuli^57^. The continuity of visual stimuli like the ones used in this study provide sufficient time to the participant to prepare a response to synchronize with the target cue^58,59^. Synchronization performance with the combined audio-visual stimulus was not significantly different from that with either of the unimodal stimuli.

For social stimuli, an opposite pattern was observed—with participants synchronising significantly better with auditory than visual stimuli. This pattern of results is consistent with the observation that the auditory modality performs better than vision in tasks that involve temporal processing^60,61^. One possibility is that this pattern of auditory dominance in synchronisation tasks is observed once the mismatch between the target stimulus and response modality is minimised. For finger tapping tasks such as the one by Hove and others^62^, the visual stimulus of a moving bar was similar to the response modality (a moving finger). Once this mismatch is minimised, the cognitive efforts could be directed entirely to the temporal aspects of the stimuli, which would result in an auditory dominance effect. In the current study, the mismatch between the target stimulus and the response modality is significantly lower for the social stimuli than the non-social stimuli, which leads to the expected pattern of auditory dominance for the social stimuli. For the main analysis, all target cues were identified auditorily, i.e. the time of peak of every *‘ba’* utterance was used to calculate the asynchrony. When visual onsets (first frame of mouth opening) were used to calculate the asynchrony, an identical pattern was observed for synchronisation with unimodal auditory and visual stimuli. Interestingly however, the synchronisation performance with the combined audio-visual stimulus differed significantly as a function of which onset type was used. When auditory onsets were used, synchronisation performance was similar to the unimodal auditory condition. However, when the visual onsets were used, performance was closer to, but significantly worse than the unimodal visual condition. This result suggests that participants tend to synchronise their verbal responses to the auditory rather than the visual cue in the audio-visual condition.

Contrary to our original hypothesis, no effect of autistic traits was noted in relation to synchronisation performance in response to either social or non-social stimuli per se. While this result is consistent with a recent report showing no autistic deficit in auditory-motor synchronisation using a finger tapping task^63^, it is in contrast to another report using coherence as a measure of synchronization of bodily movements between a live experimenter and the participant^40^. A direct comparison of the current results with these previous studies is not straightforward due to the different nature of stimuli and response modalities used. We note, however, that consistent with a previous report, a weaker social learning effect was observed in individuals with higher autistic traits^55^.

In summary, our findings suggest that humans synchronise their responses more closely with social compared with non-social stimuli. This ‘social advantage’ is likely to be driven by the preferential attention and reward linked to perceiving and interacting with other humans. Potential future research could formally examine the dependence of these results on the response modality (verbal response/ finger tapping), as well as test the impact of attention given to social compared to non-social stimuli on synchronisation performance.

## Materials and methods

### Participants and design

Fifty-three psychology undergraduates (29 females, 24 males; M_age_ = 21.01 years, range = 18–31 years) took part in the study in exchange for course credit or for cash and were screened for photosensitive epilepsy. The study had a 2 (stimulus type: social, non-social) × 3 (condition: audio, visual, audio-visual combined) within-subject design. Sample size was determined *a priori* using G*Power 3.1^64^. The analysis was based on an effect size calculated from a previously published study demonstrating improved synchronous performance to auditory compared to visual cues^25^ (*d* = *1.3*). However, since our response modality as well as stimuli type differed from the study above, we chose a more conservative effect size (d = 0.7). The analysis suggested that the minimum acceptable total sample size needed to achieve a power of .80 was 41.

### Ethics

The study was approved by the School of Psychology and Clinical Language Sciences Ethics Committee at the University of Reading. The experiment was performed in accordance with the relevant guidelines and regulations, and participants provided informed, written consent. Written informed consent was obtained to publish identifying images of the social stimulus, in an online open access publication.

### Experiment setup and apparatus

Participants synchronised their verbal responses (a *‘ba’* sound) to either audio, visual or audio-visual combined target cues. A SparkFun sound detector and an acquisition hardware (National Instruments NI-USB 6343) were used to record the presence of participants’ verbal responses. One computer was used to control and present both the visual and auditory stimuli (target cues) on the screen.

**Figure 4:**
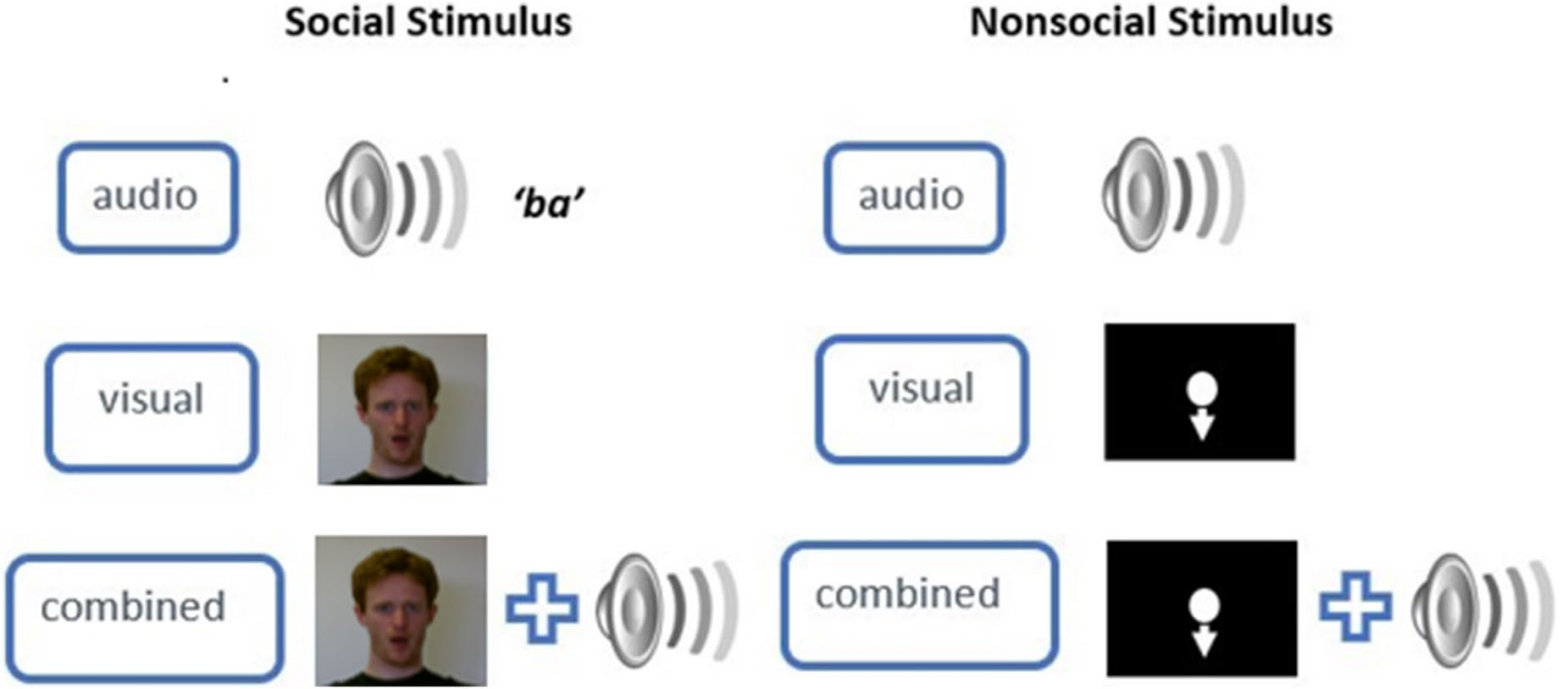
An illustration of the experimental design. Each stimulus type (social or non-social), contained audio only, visual only and audio-visual combined stimuli. For social stimuli, the audio condition consisted of a recording of a rhythmical verbal ‘*ba*’ response (either female or male), the visual condition consisted of a video recording of a female or male person performing the rhythmical ‘*ba*’ response without the presentation of the sound, and the combined condition consisted of a video recording of the female or male person performing the rhythmical ‘*ba*’ response with the sound. For non-social stimuli, either a rhythmical metronome beep (audio), a white vertically moving dot (visual) or both the metronome beep coinciding with the moving dot (combined) were presented.

Two types of target cues were presented, social and non-social (see Fig. 4). The social cues consisted of video recordings of both a male and a female actor performing a rhythmical ‘*ba*’ sound. The gender of the actor in the video recordings was matched with the gender of the participant, controlling for gender effects during synchronous activities^65^.

The actor was presented from the collar bone upwards, with a neutral facial expression, a controlled background, and wearing fitted black clothes. For visual social cue conditions, the video recording was presented without sound. Here the opening of the actor’s mouth was the target cue. For audio social conditions, a blank black screen with a white fixation cross was presented with the audio recording of the actor’s rhythmical ‘*ba*’ sound. For the male recording the ‘*ba’* sound was presented at an average of 110 Hz (minimum 108 Hz, maximum 112 Hz). Female *‘ba’* sounds were presented at an average of 211 Hz (minimum 207, maximum 215 Hz). In audio-visual social conditions, both the video and the corresponding audio recording were presented simultaneously. Non-social cues consisted of inanimate stimuli. For visual non-social condition, a white dot (2.54 cm diameter) was presented on a black background. The refresh rate of the PC monitor was 60 Hz. The dot was moving vertically with a fixed amplitude of 20 cm, using a pre-generated sine wave function. The lowest point of the downwards motion was the target cue. In the auditory non-social conditions, the generated trajectory of the dot movement was used to estimate the corresponding non-social auditory cue. The lowest peak on the x-axis for each downwards oscillation was used to generate a series of rhythmical metronome tones with a tone duration of 50 ms and at 700 Hz. In audio-visual non-social conditions, both the dot motion with its corresponding audio metronome tones were presented simultaneously (see Supplementary Material for stimulus generation code). Although there could be small discrepancies under the 10-ms range between the programmed and presented audio/visual onsets for the non-social stimuli^66^, we minimised the risk of such discrepancies by using a powerful graphics card (NVIDIA GTX 650, 4 GB), and a significantly higher screen refresh rate than the stimulus frequency. However, it should be noted that in the absence of an external photodiode to verify stimulus timings, it is not possible to quantify the magnitude of such discrepancies.

The rhythmical presentation of the target cues was varied across trials to minimise participants learning the tempo. Each trial for all conditions contained a tempo change to ensure that participants paid attention to the target cues. Six trials started with a fast, followed by a slow rhythm, and a further six trials followed the reverse order. The inter-target cue-intervals (ITI) for the fast tempo were on average 650 milliseconds (± 5%), and 870 ms (± 5%) for the slow tempo. The tempo change occurred randomly between the 5th or 7th ITI. Each condition contained 12 trials with each trial lasting 40 seconds, with an overall total of 72 trials (12 (trials) × 6 (conditions: visual social, audio social, audio-visual social, visual non-social, audio non-social, audio-visual non-social)). The presentation of both the video stimuli and the generated dot motion was controlled by Psychophysics toolbox^67^ in MATLAB (version 2014a; The Mathworks Inc., MA, USA).

The AQ was used to measure an individual’s autistic traits^44^. The AQ has 50 items measuring diverse dimensions of the autistic phenotype, such as, “I enjoy meeting new people”. Participants rate their level of agreement with each statement on a 4-point Likert scale ranging from ‘definitely agree’ to ‘definitely disagree’. Ratings are then collapsed to a yes/no scoring. Thus, the AQ scores range from 0 to 50, with autistic individuals typically scoring higher than neurotypicals. In the present study an online version of the questionnaire was administered.

### Task and procedure

Participants were asked to attend two experimental sessions, each lasting around 50 minutes, with a minimum gap of one day from one another. Each session contained either the social or non-social stimuli. The order of these sessions was counterbalanced across participants. Participants completed the AQ online when signing up for this study. For the experimental sessions, participants arrived at the laboratory individually, were greeted by the experimenter, and were seated at a table facing a PC monitor. Participants learned that the goal of the experiment was to examine the effects of different types of stimuli on their ability to verbally synchronise with them. Once participants read the information, consent was provided. In the social-stimuli session, female participants were shown the video and audio recordings of the female actor, while male participants were shown the recordings of the male actor. For visual social conditions, participants were instructed to produce a ‘*ba*’ sound in synchrony with the mouth opening of the actor presented in the video. In auditory social conditions, participants were asked to synchronise their ‘*ba*’ response in time with the ‘*ba*’ sound of the actor. For audio-visual social conditions, participants were instructed to synchronise their responses to both the audio-visual cue (‘*ba*’ sound and mouth opening) of the actor presented in the video. In the non-social stimuli session, both male and female participants were either asked to produce a ‘*ba*’ sound in synchrony with computer˗generated audio and visual stimuli. For non-social auditory conditions, participants synchronised their ‘*ba*’ response in time with an auditory metronome, whereas for non-social visual conditions participants synchronised their responses with a moving white dot at the lowest point on the vertical axis. Finally, for non-social audio-visual conditions, participants were instructed to synchronise their ‘*ba*’ responses with both the metronome and the moving dot simultaneously. The duration of each experimental session was 40 minutes.

### Analysis

The synchrony analysis adopted an information-processing approach rather than a dynamical systems approach. The latter is more favoured by researchers who examine the continuous rhythmical movements. However, in the present study participants were instructed to synchronise their verbal response to match those of an external target, rather than simply to maintain a continuous rhythm. This information processing approach has been widely used by researchers who have examined synchronous performances between two or more individuals; for example, to analyse finger tapping^68^, oscillatory arm movements^69^, bouncing^12^ and sound recording from a string quartet^70^. For each trial, we recorded the sound onsets for all verbal responses performed by participants. We then used a custom-made peak detection algorithm in Matlab to extract the onset times for each verbal response that occurred after the tempo change. Response data before and at time of the period change were excluded from the data analysis to reduce additional variability introduced due to a different starting tempo and adjustments made to entrain to a new tempo. The alignment of target and response onsets was achieved in a way where the closest response onset to the target onset was used to estimate asynchrony. For the first response onset the target onset would always precede the response onset. Missing responses were interpolated adopting methods used in previous research^71–73^. The following interpolation was conducted to account for missing responses; if a participant’s response was two times as large as the target cue’s tempo (inter-onset-interval, IOI), the participant’s inter-response-interval (IRI) was split into two (divided by two). Similarly, if a participant’s response was three times as large as the target cue’s IOI, the participant’s response was divided into three equal parts to account for the missing responses. Any responses larger than three times the relative target cue’s IOI was discarded. Absolute asynchronies were calculated to indicate the magnitude of asynchrony, irrespective of a participant being ahead or behind the target stimulus^74,75^.

**Figure 5:**
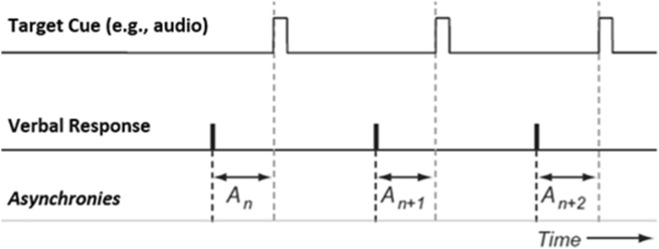
An illustration of the asynchrony calculation. Asynchrony is calculated as the difference between the event time of the respondent and the closest event time from the cue. Note, absolute asynchronies were used for the present analysis.

For non-social conditions, absolute asynchronies were calculated by calculating the difference between the target cue event time and the participant’s corresponding response event time (see Fig. 5). Non-social target cue event times were taken from the stimulus file. For non-social audio-visual combined conditions, the target onsets for both the visual and auditory cue coincided in time. The target cue event times for visual-social conditions were estimated by two independent coders in Elan^76^. The video recordings in the visual-social conditions were presented at a rate of 30 frames per second (30fps). Coders identified the first frame of mouth opening as the target event time. For audio-social conditions of the audio data from the videos was separated and saved as a wav file. The audio data was then smoothed using a bi-directional second order Butterworth low-pass filter^77^. Maximum peaks were detected using an adaptive peak detector with a threshold of a valley preceding each maximum peak. Each audio target onset event was visually cross validated with a spectrogram of the raw signal^70^. For social stimuli, it is possible to measure synchrony using either the visual or auditory onsets for the combined audio-visual conditions. We therefore examined both synchronisation performance with visual and auditory onsets for audio-visual combined conditions (see Results). However, synchronisation performance for audio-visual conditions has previously been reported to be comparable with that of the unimodal auditory conditions^20^. Therefore the audio onsets, as extracted for the audio-social conditions, were used as the target cue for audio-visual condition for the primary analysis. To explore if the use of auditory vs visual onsets had a significant impact on asynchrony, we ran a separate analysis only on the social trials (model details in the following section).

Lastly, we examined the variability of the social stimulus onsets by computing the median standard deviation across all conditions (median 0.0347 s, minimum 0.0190 s, maximum 0.220 s).

### Data reduction

Participants were excluded from the relevant analyses if one or more of these criteria were met: (a) being greater/less than 3 SD from the group mean (N = 1), and (b) missing data on a stimulus type for all conditions (N = 8). A linear mixed model analysis on the mean absolute asynchrony data was conducted from the remaining 42 participants. (see Table 1 for descriptives).

### Statistical analyses

A linear mixed model implemented in jamovi v1.1.9^78^ was defined to analyse the mean absolute asynchrony data, across all trials and after two response cycles following the tempo change in the stimulus. The model was as follows:

$$\begin{aligned} & Mean \, Absolute \, Asynchrony \, \sim \, 1 \, + \, Condition \, \left( {Auditory/Visual/Audio - visual} \right) \, \\ &\quad+ \, Stimulus \, Type \, \left( {Social/Non - social} \right) \, + AQ + Gender + Condition*StimulusType + \left( {1|Participant} \right). \end{aligned}$$

Stimulus Type (social, non-social), Condition (audio, visual or combined), Gender, AQ scores were defined as fixed effects and participants were defined as random effects. Model fit was estimated using the Restricted Maximum Likelihood (REML) method.

To check if the synchronisation performance to social combined (audio-visual) stimuli differed as a function of using auditory vs visual onsets to define the target cue, a further analysis was conducted to compare the mean asynchrony estimated by the visual and the auditory onsets respectively. This analysis was done only for trials were social stimuli were presented, since auditory and visual onsets were identical for non-social audio-visual stimuli (as programmed). The following model was estimated:

$$ \begin{aligned} & Mean \, Asynchrony \, \sim \, 1 + AQ + Condition \, \left( {Auditory/Visual/Audiovisual} \right) \,\\ &\quad + \, OnsetType\left( {Auditory/Visual} \right) \, + \, Condition*OnsetType \, + \, \left( {1|Participant} \right)\end{aligned} $$

To explore the effect of learning/practice effects through the experiment, a separate model was tested, including Trial number as a predictor. The model is specified below:

$$\begin{aligned} & Mean \, Absolute \, Asynchrony\sim \, 1 + Condition\left( {Auditory/Visual/Audio - visual} \right) \, \\ &\quad+ \, Stimulus \, Type \, \left( {Social/Non - social} \right) \, + AQ + Gender + \, Trial + Condition*StimulusType \\ &\quad+ \, StimulusType*Trial + StimulusType*Trial*AQ + \left( {1|Participant} \right). \end{aligned}$$

## Supplementary Information

Supplementary Information 1.

Supplementary Information 2.

Supplementary Information 3.
