# Supplementary material for: Keeping in time with social and non-social stimuli: Synchronisation with auditory, visual, and audio-visual cues
Source: Sci Rep. 2021 Apr 22;11:8805. doi: 10.1038/s41598-021-88112-y (PMC8062473; doi:10.1038/s41598-021-88112-y)
Supplement: Supplementary file 3 — Supplementary Information 3. [file 41598_2021_88112_MOESM3_ESM.docx]

**SUPPLEMENTARY INFORMATION**

Keeping in time with social and non-social stimuli: Synchronisation with auditory, visual, and audio-visual cues

**Juliane J. Honisch*^1^, Prasannajeet Mane^1^, Ofer Golan^2^, Bhismadev Chakrabarti*^1^**

1. School of Psychology and Clinical Language Sciences, University of Reading, Reading, RG6 6AL, UK.

2. Department of Psychology, Bar-Ilan University, Israel

**Section A – Variability of Stimuli**

The social stimuli were more variable in their timings, most likely by virtue of them being generated by humans. The median standard deviation for social stimuli across all conditions was 0.0347s (minimum 0.0190s, maximum 0.220s). The variability in the timing of non-social stimuli is likely to be significantly lower since these were generated by a computer programme (see stimulus_script.mat). While variability in the timing of non-social stimuli is likely to be minimal (Bridges et al., 2020), it is not possible to provide a quantitative estimate of this variability in the absence of an independent probe.

**Section B – Megadata.xlsx**

The data used for statistical analysis is provided in csv format. Column headings are provided in row 1. In total, there are 12 trials per condition. Each row refers to the averaged data (e.g. absolute asynchrony) per trial for each participant. The description of the column headings is described below.

*Asyncmean =* mean asynchrony calculated for each trial

*Asyncsd* = standard deviation of asynchrony for each trial

*AsyncmeanABS =* mean of the absolute asynchrony for each trial

*AsyncsdABS* = standard deviation of absolute asynchrony for each trial

*Condition=* A : audio only, V : visual only, and C : combined audio-visual

*Trial* = Trial number (note: even numbers were not used by the programme that presented the stimuli. Hence trial number 1 is the first trial, trial number 3 is the second trial, and so on).

*PP* = participant numeric identification number

*AQ* = Autism Spectrum Quotient

*S_I* = Social Interaction subscale of the AQ (factor scores using items identified by Hoekstra et al., 2008)

*A_D* = Attention to Detail subscale of the AQ (factor scores using items identified by Hoekstra et al., 2008)

*Gender =* Self-declared gender of the participant

*Stimulus Type =* indicates if the trial presented a non-social or social stimulus

*Order* = 1 indicates social stimuli were presented first and 2 non-social stimuli were presented first

**Section C – Descriptive Statistics for AQ**

Table 2 presents the descriptive statistics for AQ.

**Table 2**

**Descriptive statistics for AQ overall, and separated by gender.**

| AQ | Full Sample | Males only | Females only |
| --- | --- | --- | --- |
| Mean | 15.4 | 15.6 | 15.2 |
| S.D. | 5.22 | 5.21 | 5.22 |
| Range | 7-27 | 8-27 | 7-25 |

**Section D – Post-Hoc Analyses**

Table 1 presents a series of post-hoc t-test statistics for Stimulus Type and Condition (Bonferroni adjusted).

**Table 1**

**Post-hoc comparisons from the Stimulus* Condition interaction reported in the main analysis. (SE: standard error, t: t-statistics, df: degrees of freedom, A: Auditory, C: Combined audio-visual, V: Visual)**


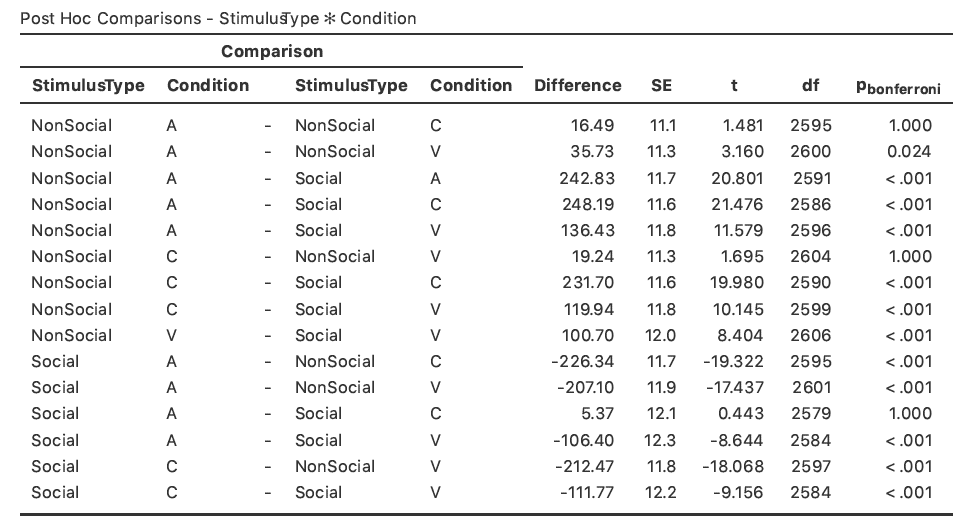


**Section E – Supplementary Analyses**

Order effects were tested through the following model:
*Mean Absolute Asynchrony ~ 1 + Condition (Auditory/Visual/Audio-visual) + Stimulus Type (Social/Non-social) +AQ+Gender+ Order+Condition*StimulusType+ StimulusType*Order+Condition*Order+(1|Participant).*
This analysis revealed no significant effect of order, or relevant 2-way interactions.

Order: F(1,39.1) = 0.639, p=0.429
Condition * Order: F(2, 2591)=0.942, p=0.390
Stimulus Type * Order: F(1,2603.7)=0.190, p=0.663
